# A rare case report of renal vein pseudoaneurysm after blunt trauma

**DOI:** 10.1097/MD.0000000000024299

**Published:** 2021-01-15

**Authors:** Yun Su Mun, Won Young Sung

**Affiliations:** a Department of Trauma Surgery; b Department of Emergency Medicine, Eulji University Hospital, Daejeon, Republic of Korea.

**Keywords:** antithrombotic agents, blunt injury, endovascular procedure, renal vein

## Abstract

**Rationale:**

Renal vein pseudoaneurysm after blunt trauma is an extremely rare clinical disease. Different interventions, such as conservative, surgical, and endovascular treatments, can be considered. However, previous studies have not described the optimal treatment strategies for this condition. Furthermore, there is a significant lack of prior case reports and of standardized treatment guidelines for trauma-induced renal vein pseudoaneurysm patients who previously maintained antithrombotic agent.

**Patient concerns:**

A 23-year-old female patient visited the emergency department after sustaining blunt injury caused by falling. The patient was diagnosed with multiple limb and rib fractures. A right renal vein pseudoaneurysm was found on abdominal computed tomography scan. Initially, there was no other organ damage, and the patient was hemodynamically stable. Thus, nonsurgical, conservative management was considered. However, the patient's hematocrit and hemoglobin levels decreased, and there was no hemodynamic improvement. The patient required lifelong treatment with aspirin because she previously underwent Fontan surgery, and orthopedic surgery for multiple fractures was planned. Thus, considering these factors, the treatment method was changed from conservative management to endovascular stent insertion.

**Diagnoses:**

Abdominal computed tomography and renal venography revealed a right renal vein pseudoaneurysm.

**Interventions:**

On the basis of the abdominal computed tomography scan and renal venography findings, the endovascular stent graft was inserted across the pseudoaneurysm area.

**Outcomes:**

Upon placement of the endovascular stent, hemoglobin and hematocrit levels gradually returned to normal. The patient's vital signs and general condition had improved. The patient recovered without any complications and was discharged 29 days after hospitalization.

**Lessons:**

Some patients with traumatic renal vein pseudoaneurysm do not experience hemodynamic improvement despite conservative treatment. Hence, endovascular procedure may be considered for these patients, particularly those who require antithrombotic treatment for a previous disease.

## 1 Introduction

Renal vascular injury is a rare complication, with an incidence rate of approximately 6% to 14%.^[1]^ Indeed, the occurrence of renal vein pseudoaneurysm after blunt trauma in the abdomen is extremely rare.^[2,3]^ Different interventions, such as conservative, surgical, and endovascular treatments, can be considered. However, previous reports have not described the optimal treatment options for this condition. Herein, we report a case of traumatic renal vein pseudoaneurysm, which was managed by changing the treatment plan from early conservative management to endovascular procedure.

## 2 Case report

**Figure 1:**
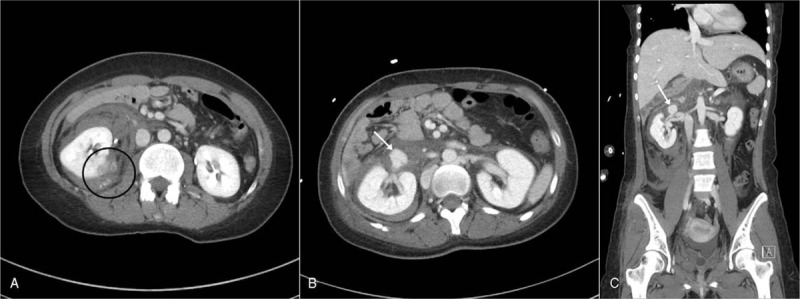
Contrast-enhanced abdominal computed tomography (CT) scan conducted in the emergency department revealed (A) a contrast leakage, which might indicate active bleeding (inner part of the black circle) in the mid-posterior part of the laceration and a hematoma in the perinephric space. Abdominal CT scan showed a pseudoaneurysm (white arrows) of the right renal vein on axial (B) and coronal (C) images.

A 23-year-old female patient who sustained injuries after falling from the fourth floor (approximately 8–9 m height) visited our emergency department. Fourteen years back, the patient underwent palliative Fontan surgery for congenital heart disease (a single ventricle with large atrial septal defect). Since then, the patient was taking aspirin daily. Despite the incident, the patient remained alert. Her vital signs were as follows: blood pressure, 133/83 mm Hg; heart rate, 99 beats/min; respiratory rate, 23 breaths/min; body temperature, 36.3°C; and oxygen saturation, 94% on room air. The initial arterial blood gas measurements on room air were as follows: blood pH, 7.42; pCO_2_ level, 30 mm Hg; pO_2_ level, 72 mm Hg; HCO_3_ level, 19.5 mEq/L; and base excess, −3.9. The patient complained of pain in the right shoulder, right flank, and right lower leg. Physical examination showed tenderness in the patient's right flank, shoulder, and inguinal region. Multi-systemic computed tomography (CT) scans were performed on this patient in order to determine the extent of the patient's injuries. Abdominal CT scan revealed right renal lacerations, with hematoma in the parenchymal and perinephric space. In the mid-posterior area with laceration, extravasation of contrast medium was suspected (Fig. 1A). Furthermore, CT scan revealed a right renal vein pseudoaneurysm (Fig. 1B, C). In addition, chest CT scan showed a thrombus in the extracardiac conduit between the inferior vena cava and pulmonary artery. The patient also sustained fractures of the right proximal humerus, right 10th and 11th ribs, inferior and superior pubic rami, and right tibia and fibula. Complete blood count analysis showed the following results: white blood cell count, 7460 cells/μL; hemoglobin (Hb) level, 13.8 g/dL; hematocrit (Hct) level, 39.6%; and platelet count, 188,000 platelets/μL. Urinalysis revealed the presence of numerous red blood cells (RBCs) per high power field.

The renal arterial angiography, which was performed due to suspected extravasation of contrast medium, did not show extravasation in the injured renal parenchyma, and vascular abnormality due to pelvic bone fracture was not observed on pelvic region angiography. As the patient presented with stable vital signs, conservative management was considered. The patient was admitted to the intensive care unit (ICU) and was monitored for delayed bleeding. On the second hospitalization day, the patient's vital signs were as follows: blood pressure, 100/62 mm Hg; heart rate, 105 beats/min. The follow-up Hb and Hct levels decreased to 10.8 g/dL and 30.1%, respectively. The patient received 2 units of packed RBCs while in the ICU. Follow-up abdominal CT scan was performed on the third hospitalization day. CT scan showed no active bleeding and significant change in the size of the right renal vein pseudoaneurysm. There was no evident active bleeding in other sites and in the renal vein pseudoaneurysm. However, the patient's Hb and Hct levels gradually decreased, and there was no improvement in blood pressure and heart rate despite blood transfusion. The patient required lifelong treatment with aspirin because she previously underwent Fontan surgery, and orthopedic surgery for multiple fractures was planned. Thus, considering these factors, the treatment method was changed from conservative management to endovascular stent insertion.

**Figure 2:**
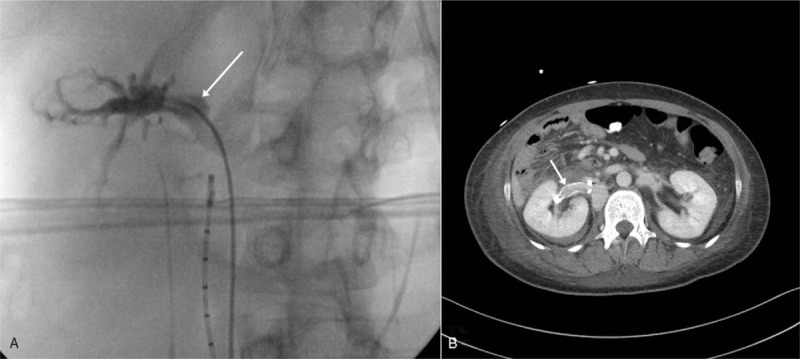
(A) C-arm fluoroscopic venography revealed the presence of a venous pseudoaneurysm as evidenced by filling of the contrast medium (white arrow). (B) Follow-up abdominal computed tomography scan performed 1 wk after stent graft insertion showed the stent in the right renal vein (white arrow) and a smaller perinephric hematoma.

On the fourth hospitalization day, the patient underwent orthopedic surgery for multiple fractures. During the induction of general anesthesia, renal venography was performed. Venography revealed the presence of a large venous pseudoaneurysm (Fig. 2A). On the basis of the abdominal CT scan and venography findings, the stent graft (12 x 40 mm size) was inserted across the pseudoaneurysm area. After several days, Hb and Hct levels gradually returned to normal. The patient's vital signs and general condition had improved. A follow-up abdominal CT scan, which was performed 1 week after the placement of stent graft, showed an intact right renal vein and a decrease in the perinephric hematoma size (Fig. 2B). After both the Hb and Hct levels normalized, aspirin and rivaroxaban were administered with consideration of the need to maintain venous stent patency and for treatment after Fontan surgery. The patient recovered without any complications and was discharged 29 days after hospitalization. However, on the basis of the opinion of a cardiologist, there is a risk of re-surgery due the presence of a thrombus in the extracardiac conduit on chest CT scan. The patient and caregiver then decided to undergo regular assessments at the hospital where the Fontan surgery was performed. Hence, we could no longer assess the patient.

## 3 Discussion

Venous pseudoaneurysm caused by trauma is an extremely rare condition.^[4–7]^ It can lead to excessive exsanguination due to rupture or thrombosis. The mechanism involved in the development of a pseudoaneurysm after blunt trauma remains unclear. However, traumatic renal vascular injury may occur due to renal vessel compression or the impact of acceleration–deceleration forces.^[1]^

Previous studies have reported about renal vascular injuries. However, most reports focused on renal arteries. To the best of our knowledge, there are only 2 studies about isolated renal vein pseudoaneurysm.^[2,3]^ Alternative treatment strategies were used in these patients. Mejia et al^[2]^ showed the possibility of conservative management in hemodynamically stable patients. Meanwhile, Monroe et al^[3]^ utilized endovascular stenting due to hemodynamic instability. Due to the limited number of case reports and the lack of established treatment guidelines, we developed a treatment strategy based on previous case reports. We initially utilized conservative treatment for a patient with a stable hemodynamic status who was admitted in the ICU. Despite this treatment regimen, the patient's Hb and Hct levels decreased, and there was no improvement in blood pressure and heart rate despite blood transfusion. A multi-systemic evaluation of our patient showed that there were no additional bleeding lesions. A follow-up abdominal CT scan revealed no significant change in the pseudoaneurysm on the right renal vein. However, due to the patient's need for lifelong treatment with aspirin and orthopedic surgery, endovascular stent grafting, which is other optional treatment, was performed.

For patients who underwent venous stent grafting, there are no currently established guidelines for preventive antithrombotic treatment and duration of drug therapy. Antithrombotic therapy is the main treatment used to prevent stent thrombosis. However, there are major discrepancies in the use of antithrombotic agents based on previous studies.^[8]^ Data showed that aspirin is effective in preventing arterial stent thrombosis. However, whether it can prevent venous stent thrombosis remains unclear. Antiplatelet therapy alone did not change the patency rate from follow-up to venous stenting based on a systemic review.^[9]^ Another study showed that concomitant antiplatelet and anticoagulation therapy is more effective in maintaining stent patency after iliocaval venous stenting than anticoagulation therapy alone.^[10]^ On the basis of the results of previous studies and with consideration that the patient required lifelong treatment with aspirin after Fontan surgery, we used rivaroxaban (direct oral anticoagulant) in combination with other treatments because it is easy to use and does not require monitoring.

Despite advances in diagnosis and treatment over the previous years, severe renal injury still has an inherent risk of death. One study showed that the kidney can be preserved after major renal vascular injury in 25% to 35% of cases.^[1]^ Nonsurgical conservative treatments are important in reducing nephrectomy and preserving renal function. In relation to this, effective and safe interventions for major renal injuries are still developed. The placement of an endovascular stent should be primarily considered for the treatment of traumatic renal vein pseudoaneurysm in hemodynamically unstable patients. Previous case reports of this condition are limited. Furthermore, there is a lack of standardized treatment guidelines for the management of trauma-induced renal vein pseudoaneurysm in patients who previously received maintenance treatment with antithrombotic agents. However, some patients do not experience improvement in hemodynamic status despite conservative management. Hence, if continuous antithrombotic therapy is required for the management of a previous disease in these patients, endovascular stent graft should be considered as another optional treatment plan.

## Author contributions

**Conceptualization:** Yun Su Mun, Won Young Sung.

**Supervision:** Won Young Sung.

**Writing – original draft:** Yun Su Mun, Won Young Sung.

**Writing – review & editing:** Yun Su Mun, Won Young Sung.

## Abbreviations

Abbreviations: CT = computed tomography, Hb = hemoglobin, Hct = hematocrit, ICU = intensive care unit, RBC = red blood cell.
